# Takotsubo syndrome as initial presentation of pheochromocytoma in young children: case report and literature review

**DOI:** 10.1186/s12887-025-06187-2

**Published:** 2025-10-11

**Authors:** Enfu Huang, Ting Zhang, Xiangming Yan, Ling Sun, Yun Zhou

**Affiliations:** 1 Department of Urology, Children’s Hospital of Soochow University, Suzhou, Jiangsu P.R. China; 2 Department of Cardiology, Children’s Hospital of Soochow University, Suzhou, Jiangsu P.R. China

**Keywords:** Takotsubo syndrome, Pheochromocytoma, Heart failure, Case report

## Abstract

**Background:**

Takotsubo syndrome is an acute and reversible form of left ventricular dysfunction most often seen in postmenopausal women and typically triggered by extreme emotional or physical stress. Its occurrence in children is exceedingly rare. Pheochromocytoma, a catecholamine-secreting tumor, can mimic Takotsubo syndrome due to its cardiovascular effects. This case is reported for its rarity and to raise awareness of pheochromocytoma as a potentially life-threatening but reversible cause of stress cardiomyopathy in children.

**Case presentation:**

A 7-year-old boy presented with signs of acute heart failure, including respiratory distress and hypertension. Echocardiography revealed severe left ventricular dysfunction, necessitating invasive mechanical ventilation and high-dose inotropic support. Initial cardiac magnetic resonance imaging and laboratory evaluations were inconclusive. Persistent hemodynamic instability and unexplained cardiac dysfunction prompted a multidisciplinary evaluation. Abdominal magnetic resonance imaging revealed an adrenal mass, and biochemical analysis confirmed elevated catecholamines, leading to a diagnosis of pheochromocytoma. Management included alpha-adrenergic blockade to control hypertension and correct volume status, followed by cautious initiation of beta-blockade. Despite medical therapy, the patient’s cardiac function did not improve significantly, and surgical resection of the adrenal tumor was performed on hospital day 10. Histopathological examination confirmed the diagnosis of pheochromocytoma. Postoperatively, cardiac function improved rapidly.

**Conclusions:**

This case highlights pheochromocytoma as a rare but important differential diagnosis in pediatric patients presenting with acute heart failure and Takotsubo-like cardiomyopathy. Early recognition and targeted management can lead to complete recovery. Increased awareness among pediatricians and cardiologists is essential to avoid delayed diagnosis and improve outcomes in similar cases.

## Background

Takotsubo syndrome (TTS) was first described in 1990 as a rare condition characterized by transient ventricular wall akinesia in the absence of significant coronary artery obstruction. The proposed mechanism involves catecholamine surge, typically triggered by severe emotional or physical stress, leading to reversible myocardial dysfunction [1–3]. 

Pheochromocytoma, a catecholamine-secreting neuroendocrine tumor, has an estimated incidence of 1/300,000 individuals annually [4]. Pediatric cases account for approximately 10% of all pheochromocytomas, with a median age at diagnosis of 11–13 years and a male predominance (male: female ratio = 2:1) [5, 6]. More than 90% of pheochromocytoma arise intra-abdominally and most frequently in the adrenal. Pheochromocytoma typically presents with paroxysmal or refractory hypertension due to catecholamine excess. Takotsubo-like cardiomyopathy can be associated with pheochromocytoma, but is rare in the pediatric population. We report a prepubertal case of pheochromocytoma that initially presented as Takotsubo-like cardiomyopathy.

## Case report

**Fig. 1:**
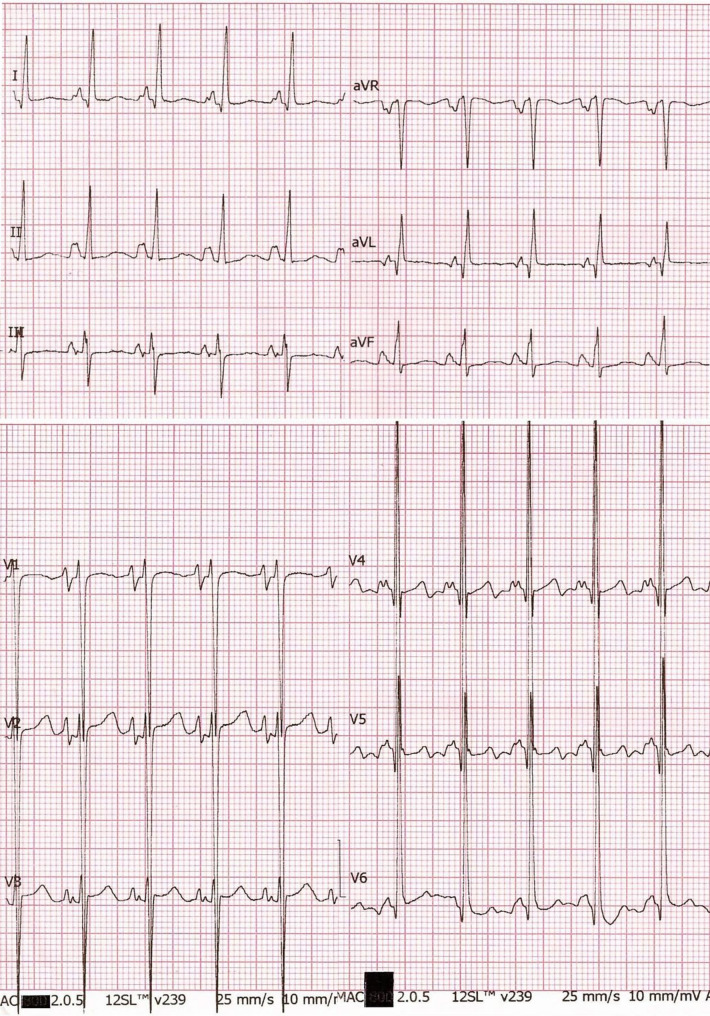
ECG shows sinus tachycardia(126 bpm); Left ventricular and left atrium hypertrophy; ST-T elevation in V1-V3 and ST-T change in V4-V6

**Fig. 2:**
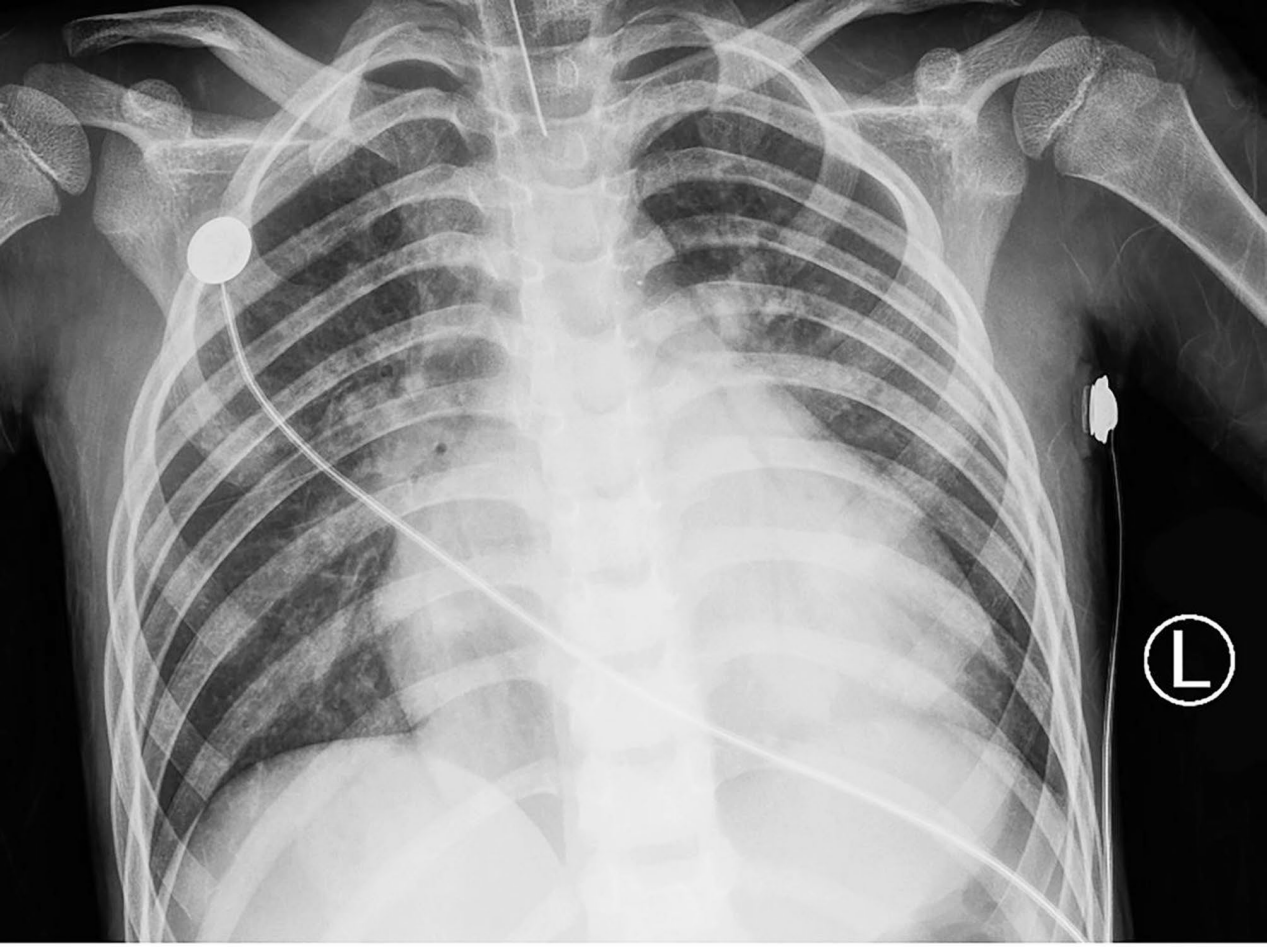
Chest X-ray shows permeability was reduced in bilateral lung field and enlargement of cardiac silhouette (CTR=0.68)

A previously healthy 7-year-old male presented to the emergency department with severe hypodynamic shock, profuse sweating, and intense thirst. His symptoms began one month prior following an upper respiratory tract infection, with progressive fatigue despite unremarkable outpatient evaluations. On presentation, the electrocardiogram (ECG) revealed ST segment elevation in V1-V3 and ST changes in V4-V6 (Fig. 1). Blood tests showed leukocytosis, elevated troponin T and BNP, and signs of decompensated metabolic alkalosis. Chest X-ray indicated increased lung markings and cardiomegaly (cardiothoracic ratio = 0.68) (Fig. 2).

**Fig. 3:**
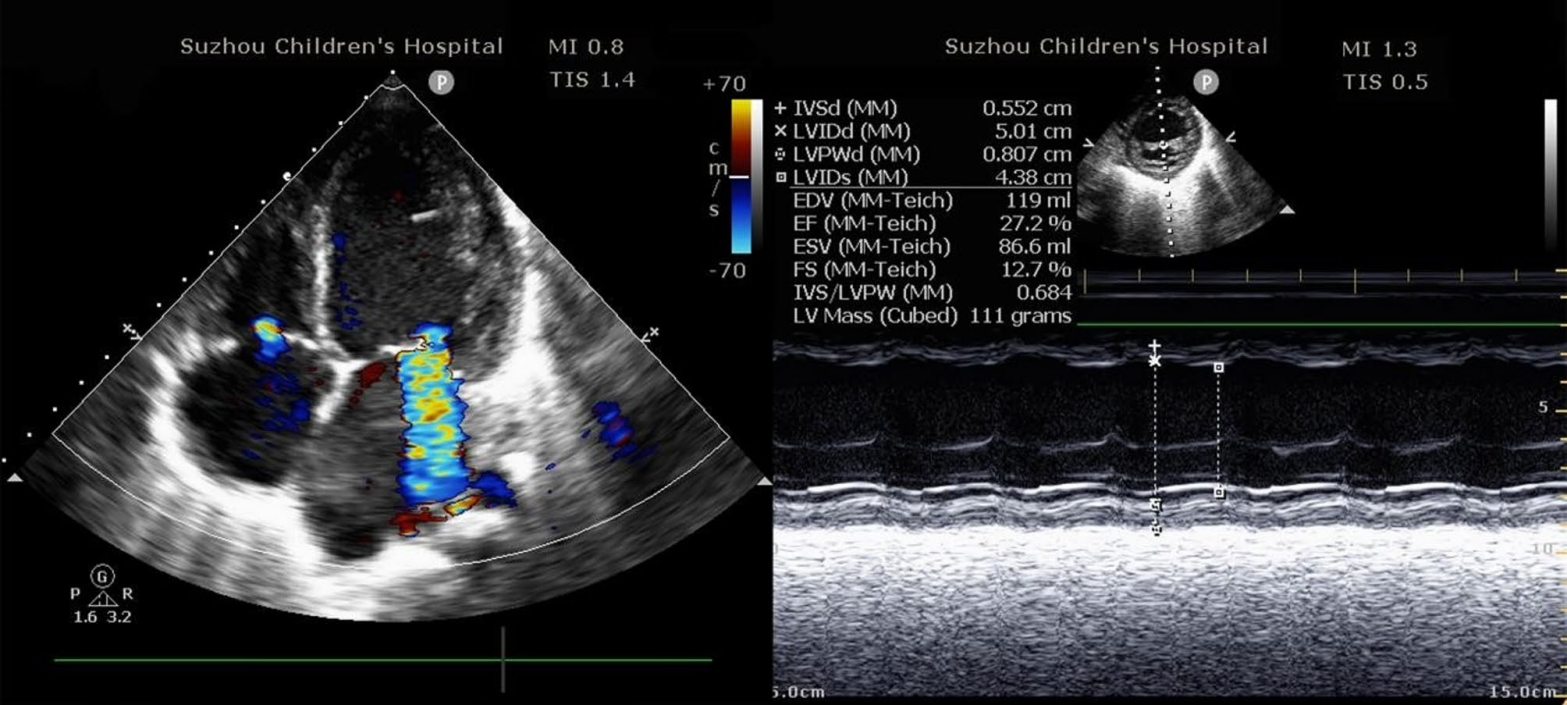
Dilated left ventricle and paradoxical LV movement on M-mode echocardiography

Upon admission to the Intensive Care Unit (ICU), his vital signs showed tachycardia (139 bpm) and hypertension (140/102 mmHg). Ultrasound cardiac output monitoring (Uscom) indicated low-displacement, high-obstruction cardiac insufficiency. Echocardiography revealed an enlarged left ventricle with a reduced ejection fraction (LVEF = 27%, FS = 13%, LVIDd = 50.1 mm, z score = 4.6) [7] (Fig. 3). The patient was treated with milrinone, methylprednisolone hemisuccinate, and albumin to improve cardiac contractility and manage inflammation. Four hours after admission, tracheal intubation was performed due to polypnea and cold, moist extremities. The patient was initially diagnosed with acute decompensated heart failure and chronic cardiac insufficiency. Despite treatment, hypertension and tachycardia persisted.

**Fig. 4:**
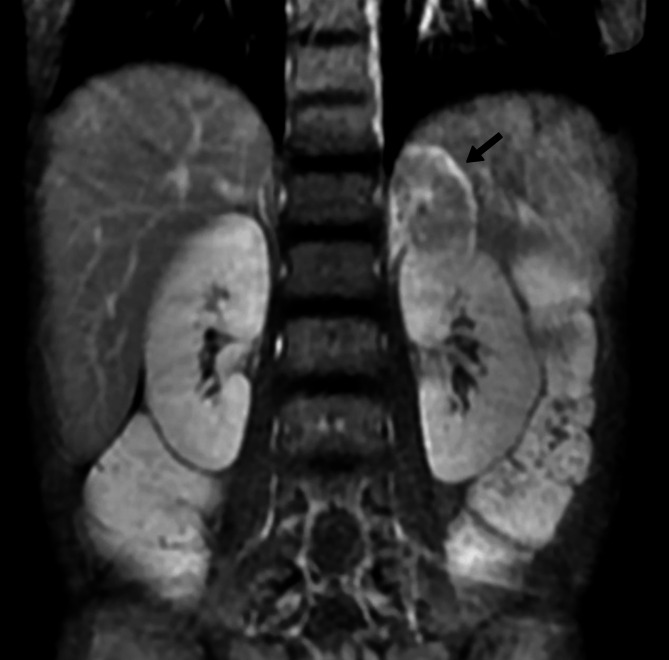
Abdominal magnetic resonance imaging(MRI) demonstrates 39mmx38mx29mm round shape and a mixture of high- and low-density area(arrow) above the left kidney. There seemed to be no infiltration into surrounding organs

**Fig. 5:**
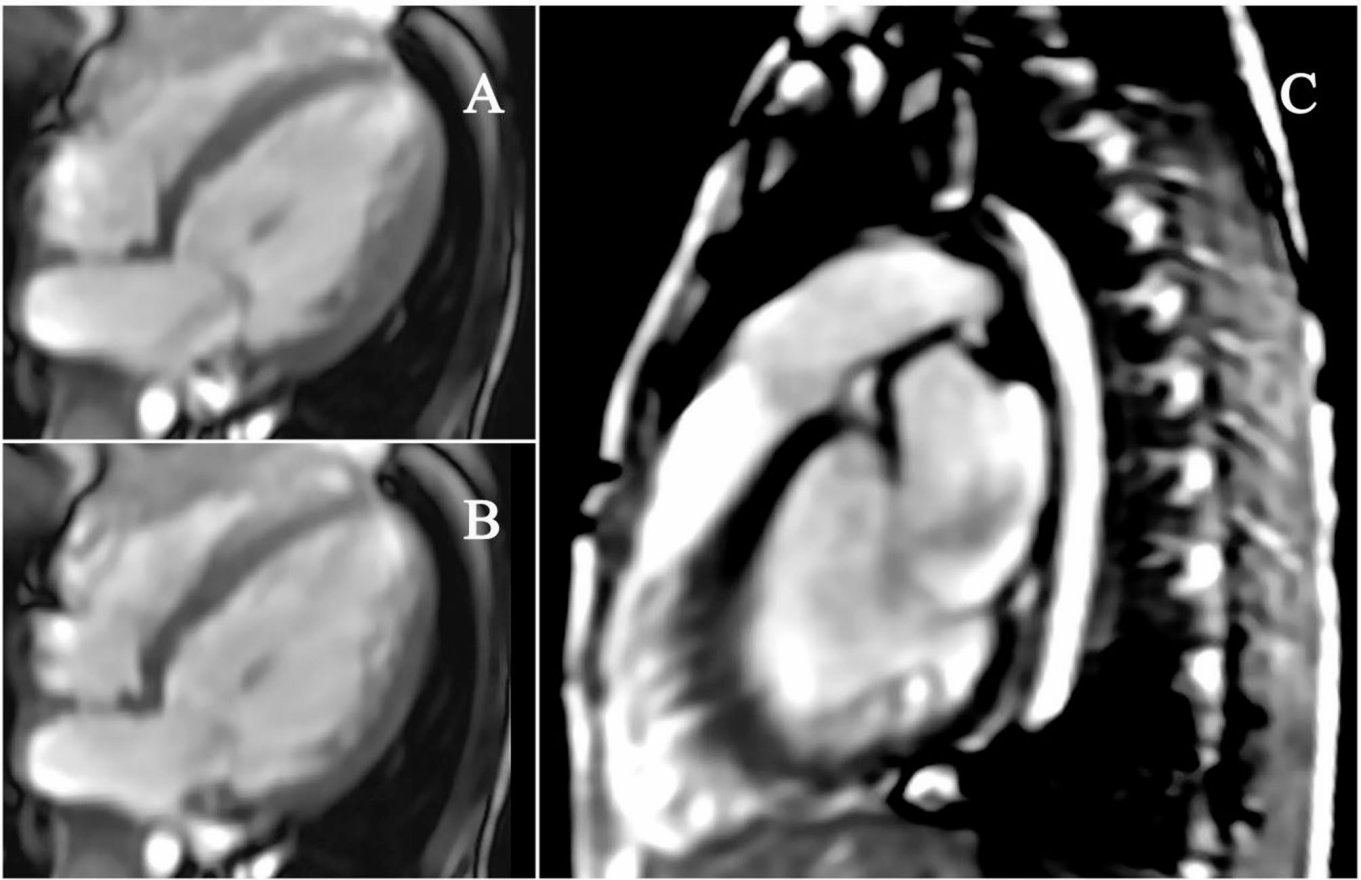
Cine sequences of cardiac magnetic resonance imaging(MRI) during systole(**A**) and diastole(**B**) in the acute phase. Ontrast-enhanced cardiovascular MRI did not show myocardial delayed hyperenhancement(**C**)

Follow-up echocardiography revealed no evidence of coronary artery obstruction or apical ballooning. Importantly, the persistence of hypertension—an atypical finding in pediatric decompensated heart failure—served as a pivotal diagnostic clue that shifted the focus toward secondary etiologies. This prompted a targeted evaluation, during which abdominal ultrasonography identified a well-circumscribed 40 × 40 × 30 mm mass in the left adrenal gland. Subsequent cross-sectional imaging, including CT and MRI, was highly suggestive of a pheochromocytoma (Fig. 4), while cardiac MRI revealed no delayed hyperenhancement (Fig. 5). 24-hour urine vanillylmandelic acid (VMA) levels (20 mg/24 h, normal ≤ 12 mg/24 h), and plasma catecholamines (norepinephrine 120039.0 pmol/L, normal: 1182.8-10054.0 pmol/L; epinephrine 142.9 pmol/L, normal ≤ 769.0 pmol/L) were significantly elevated.

The patient was treated with phentolamine and milrinone, in combination with sodium nitroprusside, to control blood pressure and enhance cardiac function. After ten days, nifedipine and captopril were added for blood pressure management, alongside hypervolemic therapy. A slight improvement in left ventricular ejection fraction (LVEF = 38%, FS = 18%, LVIDd = 54.1 mm, z score = 5.7) [7] was noted, but persistent tachycardia (121/min) and hypertension (125/78mmHg) led to a decision for surgical resection of the mass. Preoperative management included sodium nitroprusside, phentolamine, esmolol, and milrinone to stabilize the patient’s hemodynamics.

**Fig. 6:**
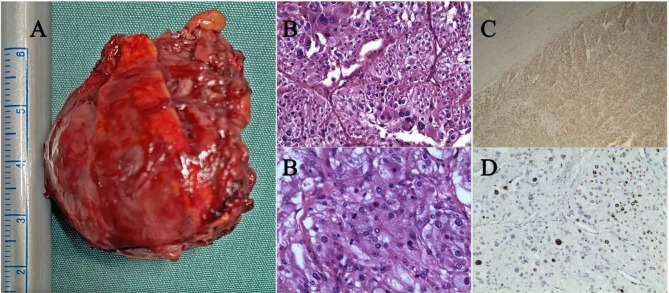
Macroscopic aspect of the left adrenal tumor(**A**). Specimens stained with hematoxylin and eosin showing pheochtomocytoma(**B**). The immunohistochemistry confirmed positive cytoplasmic staining for chromogranin A(**C**) and Ki-67(**D**)

The tumor was successfully removed via retroperitoneal laparoscopy in 40 min. Intraoperative hypotension (BP 80/50 mmHg) was managed with volume resuscitation and norepinephrine infusion. Pathological examination confirmed pheochromocytoma (Fig. 6). Postoperatively, milrinone and norepinephrine infusions were continued in the ICU. Four days after surgery, plasma norepinephrine levels decreased to 2538.5 pmol/L. Four months postoperatively, cardiac function had significantly improved (EF = 68%, FS = 38%, LVIDd = 46.2 mm, z score = 2.8) [7].

## Discussion

TTS in children is rare, and when secondary to pheochromocytoma (PHEO), even rarer. Catecholamine surges from tumors can induce coronary vasospasm, microvascular dysfunction, and myocardial injury, ultimately resulting in a Takotsubo-like cardiomyopathy (TLC) [8–10]. In 2007, the Takotsubo Cardiomyopathy Study Group had recognized PHEO as a known cause of catecholamine-induced TLC [9]. With an increased understanding of the PHEO-induced Takotsubo-like cardiomyopathy (PHEO-TLC), the number of case reports has increased in recent years. Although pheochromocytoma may lead to myocardial abnormalities, cardiomyopathy is rarely reported in children than in adult patients.

Mechanistically, catecholamine surges disrupt β-adrenergic signaling, impair calcium homeostasis, and damage mitochondria, leading to reduced ATP synthesis and contractile dysfunction. Emerging evidence also implicates the brain–heart axis, highlighting a central neurocardiogenic response in TTS. Regardless of the mechanism, catecholamine-induced cardiac dysfunction may mimic acute myocarditis or heart failure, often delaying diagnosis in children [11]. In our case, extremely elevated plasma catecholamine levels likely accounted for the patient’s initial cardiac dysfunction.

We conducted a computer-assisted search through PubMed and identified 10 pediatric patients from 9 published case reports (Table 1) [12–20]. By reviewing and synthesizing these cases alongside our own, we aim to highlight the need for more precise characterization of symptom profiles in affected children, to better define the clinical spectrum of PHEO-TLC.

**Table 1:** patients, presenting symptoms, electrocardiographic & ECHO abnormalities, plasma or urinary catecholamine, and the prognosis

Author (year)	Age (year) (sex)	Clinical features at presentation	● Blood pressure ● Heart rate ● Respiration (time of admission)	ECG findings	ECHO/ cardioangicgraphy	plasma/ urinary catecholamine & metabolites
M.S. Schaffer (1981) [12]	11 (M)	● Abdominal pain; ● Anorexia; ● Shortness of breath;	● 110/80 mmHg ● 128 bpm ● Unknow	● Left axis deviation; ● Left ventricular hypertrophy with strain;	● None;	None
M.S. Schaffer (1981) [12]	8 (F)	● Abdominal pain; ● Dyspnea/shortness of breath; ● Progressive exercise intolerance; ● Athrepsy;	● 110/50 mmHg ● 136 bpm ● 32 breath/min	● Sinus tachycardia; ● Left atrial enlargement; ● Left ventricular hypertrophy; ● ST elevation ross the midprecordium;	● LVEF = 15%;	urinary VMA: (+)
M.I. Dagartzikas (2002) [18]	13 (M)	● Sudden loss of consciousness; ● Anxious; ● Diaphoretic;	● 160/104 mmHg ● 120 bpm ● 30–40 breath/min	● None	● LVEF = 12%; ● Left-sided mural thrombus;	None
H. Nicholas (2009)	17 (M)	● Vomiting of frothy & bloody material; ● Trouble keeping balance ● Hypertensive;	● 150–160/-mmHg; ● 190 bpm ● Unknow	● Anterior infarction; ● Deep Q-waves; ● ST changes(V1-V6);	● LVEF = 32%; ● Inferior wall, apical & anterior septal dyskinesis;	Blood NE: (+)
N.Narin (2011) [15]	16 (M)	● Abdominal pain; ● Nausea; ● Dizziness; ● Diaphoretic; ● Alert consciousness;	● 80/60 mmHg ● 160 bpm ● Unknow	● Sinus tachycardia; ● ST-T changes; ● Long QT;	● LVEF = 38%; ● Mild mitral regurgitation; ● Aortic regurgitation;	urinary metamephrine & NE: (+);
Y.Kalra (2012) [17]	9 (M)	● Fever; ● Lethargy; ● Increased work of breathing;	● 120/77 mmHg ● 110 bpm ● Unknow	● Right bundle branch block	● Left ventricular dysfunctionleft Ventricular dilation; ● Left ventricle thrombus;	urinary VMA: (+)
F. Santoro (2012) [20]	17 (F)	● Abdominal pain; ● Headache; ● Psychomotor agitation; ● Nausea & vomiting;	● 170/140 mmHg ● 170 bpm ● Unknow	● Sinus tachycardia; ● ST elevation (V3–V6,DII, DIII, aVF)	● LVEF = 20%; ● Apical ballooning;	urinary noradrenaline: (+)
H. Lam (2014) [16]	9 (M)	● Fever; ● Lethargy; ● Abdominal pain;	● Unknow ● Unknow ● Unknow	● None	● LVEF = 27%; ● Left ventricle thrombus;	VMA & NE: (+)
S.W. Sharkey (2015) [19]	16 (M)	● Chest pain; ● Light-headedness; ● Nausea;	● 114/47 mmHg ● 126 bpm ● Unknow	● Sinus tachycardia; ● ST elevation(V2,V3)	● LVEF = 52%; ● Regional wall motion abnormality; ● Mid-LV Segments dyskinesis; ● Base and apex hyperkinesis;	Plasma & urinary metamephrine & NE: (+);
S.A. Uçaktürk (2020) [14]	12 (M)	● Vomiting; ● Palpitations;	● 113/81 mmHg ● 125 bpm ● Unknow	● Sinus tachycardia	● LVEF = 48%; ● Mild mitral regurgitation; ● Aortic insufficiency;	urinary metamephrine & NE: (+);

Most pediatric TTS cases reported have a median age of 8–9 years, while the pediatric PHEO-TLC cases have a median age of approximately 12.5 years, aligning more closely with the typical age distribution of pediatric PHEO [21, 22]. Our patient, at 7 years old, may represent the youngest reported case in the English literature to date. Due to the rarity of the condition, the precise age distribution remains uncertain. Unlike adult TTS, which predominantly affects females, the pediatric cases of PHEO-TLC show a more balanced gender distribution, likely reflecting the higher prevalence of pheochromocytoma in male patients [8]. 

Across these cases, tachycardia (90%), gastrointestinal symptoms (80%), general weakness (40%), and respiratory complaints (40%) were frequently reported. Notably, the classic triad of pheochromocytoma—paroxysmal headache, sweating, and palpitations — was absent, consistent with literature suggesting this triad is less common in adrenergic cardiomyopathy. Sustained hypertension, rather than the classic symptom triad, is often the most consistent clinical feature and should prompt further investigation when present [23]. In our case, the patient initially presented with a one-month history of progressive fatigue and profuse sweating, along with elevated myocardial enzymes and electrocardiographic abnormalities. These findings led to a preliminary diagnosis of acute decompensated heart failure, with chronic cardiac insufficiency of unclear origin also considered. However, despite the absence of the classic triad, the persistence of hypertension raised clinical suspicion and prompted further evaluation. Abdominal ultrasonography subsequently revealed a left adrenal mass.

In children, acute cardiac insufficiency is usually attributed to congenital heart disease, myocarditis, or acute exacerbation of chronic heart failure [24]. And most patients with pheochromocytoma exhibit abnormal findings on ECG, as well as elevated levels of B-type natriuretic peptide(BNP) and N-terminal proB-type natriuretic peptide(NT-proBNP), but typically less pronounced than in acute cardiac insufficiency [25–27]. Therefore, when the above conditions occur in patients along with hypertension in patients without congenital structural abnormalities, should be alarming.

Our case highlights the diagnostic challenges of PPGL-TLC in children. The initial presentation chronic cardiac insufficiency and upper respiratory infection, only after observing persistent hypertension did abdominal imaging reveal an adrenal mass. Pediatric pheochromocytoma should be considered in children with unexplained hypertension and signs of cardiac dysfunction.

Crucially, beta-blockers must not be administered before initiating adequate alpha-blockade, as unopposed alpha stimulation may precipitate hypertensive crisis or circulatory collapse. Medical management should proceed with high clinical suspicion and biochemical confirmation [27]. In this case, the cardiology team’s appropriate pharmacologic management played a pivotal role in stabilizing the patient’s cardiovascular status and facilitating safe preoperative preparation.

Adrenalectomy remains the only definitive treatment for pheochromocytoma. In our case, multidisciplinary collaboration—particularly with cardiology—was instrumental in identifying the underlying etiology and ensuring perioperative stability. Most of the literature we can find about PHEO-TLC didn’t mention the preoperative blood pressure, except for one case of hypotension on rare occasions. In contrast, part of the literature describes poor blood pressure control before surgery. In our review, five cases did not meet the international consensus recommendations for preoperative optimization. Due to the absence of pediatric-specific guidelines for the treatment of pheochromocytoma, we referred to adult guidelines and international consensus, which advise 7–14 days of pharmacologic preparation to achieve hemodynamic goals [28, 29]. Specifically, target blood pressure should be below the 90th percentile for age, and heart rate should fall between the 10th and 90th percentiles based on age-based reference charts. This consensus also note that longer durations may be necessary in pediatric patients. Nonetheless, even with alpha-blockade, perioperative hemodynamic instability remains common. Some studies have further suggested that prolonged alpha-blockade may increase the risk of intraoperative hypotension [30]. More recently, evidence has emerged indicating that in selected cases, pheochromocytoma surgery may be safely performed without routine preoperative pharmacologic preparation, without significantly increasing complications.

While we recognize the importance of achieving hemodynamic stability to reduce intraoperative fluctuations and the risk of cardiogenic shock, we recommend proceeding with surgery in pediatric patients who fail to achieve target blood pressure and heart rate after more than two weeks of medical management, in order to prevent irreversible cardiovascular complications.In our case, involvement of a multidisciplinary team—including the pediatric cardiologists—was instrumental in identifying the underlying etiology and ensuring perioperative stability.

## Conclusion

Pheochromocytoma should be considered in pediatric patients with unexplained hypertension and cardiac dysfunction. Early recognition and multidisciplinary management are essential. While optimal preoperative hemodynamic control remains ideal, surgical intervention should not be unduly delayed when medical management proves insufficient.

## Data Availability

All case-specific data (including clinical records, imaging studies, and laboratory findings) are presented within the manuscript or supplementary materials. The use of raw data (e.g., original medical records or imaging files) was authorized by the patient’s guardians through written informed consent, and all data were anonymized. De-identified data may be shared upon reasonable request and ethics approval by contacting the corresponding author(Email: zhangting0614@suda.edu.cn) for further inquiries.Data for literature review were extracted from publicly available sources (PubMed, Web of Science, and CNKI), with search strategies (keywords, inclusion/exclusion criteria) detailed in the Methods section. All cited references are listed in the bibliography. Additional literature analysis datasets can be accessed via the mentioned databases or requested from the authors.

## Abbreviations

TTS: Takotsubo syndrome
MRI: Magnetic resonance image
PCCs: Pheochromocytomas
CT: Computed tomographic
TLC: Takotsubo-like cardiomyopathy
PHEO-TLC: Pheochromocytomas-induced Takotsubo-like cardiomyopathy
